# Computational development of a molecular-based approach to improve risk stratification of endometrial cancer patients

**DOI:** 10.18632/oncotarget.25354

**Published:** 2018-05-22

**Authors:** Federica Torricelli, Davide Nicoli, Riccardo Bellazzi, Alessia Ciarrocchi, Enrico Farnetti, Valentina Mastrofilippo, Raffaella Zamponi, Giovanni Battista La Sala, Bruno Casali, Vincenzo Dario Mandato

**Affiliations:** ^1^ Laboratory of Translational Research, Azienda USL Reggio Emilia-IRCCS, Reggio Emilia, Italy; ^2^ Laboratory of Molecular Biology, Azienda USL Reggio Emilia-IRCCS, Reggio Emilia, Italy; ^3^ Department of Electrical, Computer and Biomedical Engineering, University of Pavia, Pavia, Italy; ^4^ Unit of Surgical Gynecologic Oncology, Azienda Unità Sanitaria Locale-IRCCS di Reggio Emilia, Reggio Emilia, Italy; ^5^ Unit of Obstetrics and Gynaecology, University of Modena and Reggio Emilia, Reggio Emilia, Italy; ^6^ Unit of Obstetrics and Gynaecology, Azienda Unità Sanitaria Locale-IRCCS di Reggio Emilia, Reggio Emilia, Italy

**Keywords:** endometrial cancer, somatic mutations, prognosis, classification tree, next generation sequencing

## Abstract

Histological classification and staging are the gold standard for the prognosis of endometrial cancer (EC). However, in morphologically intermediate and doubtful cases this approach results largely insufficient, defining the need for better classification criteria.

In this work we developed an algorithm that based on EC genetic alterations and in combination with the current histological classification, improves EC patients prognostic stratification, in particular in doubtful cases. A panel of 26 cancer related genes was analyzed in 89 EC patients and somatic functional mutations were investigated in association with different histology and outcome.

An unsupervised hierarchical clustering analysis revealed that two groups of patients with different tumor grade and different prognosis can be distinguished by mutational profile. In particular, the mutational status of APC, CTNNB1, PIK3CA, PTEN, SMAD4 and TP53 resulted to be principal drivers of prognostic clustering. Consistently, a decisional tree generated by a data mining approach summarizes the consequential molecular criteria for patients prognostic stratification.

The model proposed by this work provides the clinician with a tool able to support the prognosis of EC patients and consequently drives the choice of the most appropriated therapeutic strategy and follow up.

## INTRODUCTION

Endometrial cancer (EC) is the most common gynecological cancer in industrialized countries. About 142000 new cases of EC are diagnosed every year worldwide, and about 42000 women die every year from EC [1]. Most ECs are diagnosed after the menopause, with the highest incidence around the seventh decade of life [1]. The early onset of symptoms explains why, at the time of the diagnosis, 70% of the patients present an early-stage disease, thus far resulting in a favorable prognosis with 77% 5-year overall survival rate (OS). On the contrary, women with advanced or recurrent disease present a low response rates to conventional chemotherapy and extremely poor outcomes [2].

Traditionally, EC is classified into two types according to Bokhman model based upon clinical-pathologic features [3]. Type 1 ECs are endometrioid cancer, associated with hyperestrogeneism and typically preceded by endometrial hyperplasia. They are often diagnosed at an early stage, and have a good prognosis. Type 2 EC includes non-endometrioid cancers such as serous, clear cell, mixed cell, undifferentiated and carcinosarcoma. These neoplasms not estrogens correlated, often occur in the presence of an atrophic endometrium and have a poor prognosis. The 5-year OS rate of patients with endometrioid adenocarcinoma (type 1) range from 75% to 86%, in contrast to 50% to 60% of patients with non-endometrioid cancer (type 2).

Genetically, Type 1 endometrioid ECs present high percentage of mutations in PTEN, KRAS, ARID1A and CTNNB1, as well as defects in DNA mismatch repair. Type 2 non-endometrioid ECs frequently show aneuploidy, p53 mutations and HER2 amplification. PIK3CA mutations are frequent in both EC histotypes [4]. Well known prognostic factors are age, International Federation of Gynaecology and Obstetrics (FIGO) stage, depth of myometrial invasion, tumor differentiation grade, tumor type and lymphovascular space invasion (LVSI) [5, 6]. Moreover, new prognostic factors were investigated [7–9] to identify tumor with poor outcome. Although more than one risk-based classification of EC have been proposed numerous EC cases, in particular those with intermediate phenotype and grading (e.g. endometrioid tumor G2) still have uncertain prognosis.

Recently, The Cancer Genome Atlas Research Network (TCGA) reported a comprehensive genomic and transcriptomic analysis of EC based on next-generation sequencing (NGS) technologies, analysis of DNA methylation, reverse-phase protein array, and microsatellite instability [10]. The study categorized the most common histotypes into four genomic classes: ultra-mutated tumors (POLE) with a favorable prognosis, microsatellite-instable tumors (microsatellite hyper-mutated) and low copy number tumors (microsatellite-stable) both with an intermediate prognosis and high copy number tumors (serous-like) with a poor outcome. Moreover the TCGA study revealed also that a subset of ECs diagnosed as high-grade endometrioid carcinomas harbored copy number and mutational profiles more similar to those of serous ECs and in general no mutations (excluding POLE) were identified as unique to any of the four genomic classes. In view of the substantial genetic and morphological heterogeneity in EC, these data suggested that the current histopathology-based classification approach requires a revision, which could take into account also the complicated molecular profiles of EC [4]. While offering a complete overview of the EC genetic and molecular landscape, the TCGA classification was only partially associated with prognosis, giving results that seem to be in contrast with literature data and would need further investigation. Furthermore, the use of this type of screening in clinical routine, where a rapid prognostic prediction and treatment choice it’s needed, appears still not feasible because too expensive in terms of time, cost and interpretation of the results, due to its elevated complexity.

In this study we investigated a novel molecular-based approach to predict prognosis in EC. The model, based on DNA sequencing of few genes, subdivides EC in “good prognosis” and “bad prognosis“ and can be applied in the investigation of ambiguous cases, and to support Bokhman’s model and histological grading when the canonical approach is not sufficient to predict tumor outcome.

## RESULTS

### Study population

In this study 89 EC patients were analyzed. Clinical and pathological features of the patients enrolled in this study are shown in Supplementary Table 1. Mean age of this cohort was 65 years (range 42–85 years) and mean BMI was 32 (range 19–59). Fifty patients had hypertension and 19 were affected by diabetes. Eighty-two patients presented endometrioid type 1 EC while the remaining 7 patients had type 2 EC. Among the 82 type 1 tumors, 33 had a well differentiated G1 histotype, 16 had a G2 histotype and 33 patients had an undifferentiated G3 histotype. According to FIGO classification, 13 tumors were staged as IA, 36 as IB, 17 as IC; eight patients were staged as II, 14 as III (5 IIIA, 1 IIIB and 8 IIIC) and one as IV. In compliance with Lax Kurman classification, 49 cases were low grade, 36 were high grade and 4 cases remained unclassified.

Mean follow up was 79 months (range 6–192 months), 14 patients had a recurrence during follow up (14/89, 15.7%) and 11 patients died because of the tumor (11/89, 12.3%).

### Next generation sequencing analysis revealed a massive genetic mutations frequency in endometrial cancer population

A next generation sequencing approach was applied to investigate mutations in a panel of 26 oncogenes and onco-suppressors genes. In order to outline a molecular profile with prognostic potential we considered only genetic variants known to have a frequency lower than 1% in total population and supposed to have an effect on protein coding. Supplementary Table 2 summarizes all 893 genetic alterations identified by sequencing. Variants classified as synonymous, intronic, non coding, polymorphic or localized in 3’UTR regions (608) were excluded while variants classified as missense, frameshift, stop-gain or affecting splicing sites (285) were included in further analysis. Seventy-six of 89 ECs (85.4%) presented at least one somatic mutation in one of the 26 genes analyzed while 13 ECs (14.6%) didn’t present any somatic mutation in the considered genes. PTEN and PIK3CA resulted the most mutated genes: 56/89 (62.9%) patients presented at least one mutation in PTEN, 37/89 (41.6%) in PIK3CA. 20 patients had more than one somatic mutation in PTEN and 12 had more than one in PIK3CA. Even 4 mutations for gene in the same patient were identified for PIK3CA and PTEN.

Thirteen genes (APC, BRAF, CTNNB1, EGFR, FGFR2, FBXW7, KRAS, MET, NRAS, PIK3CA, PTEN, SMAD4, TP53) were mutated in at least 5 patients. Five genes (AKT1, ALK, CDH1, GNAS, PDGFRA) were mutated in only one patient. Genes APC, CTNNB1, EGFR, FBXW7, KRAS, MET, NRAS, PDGFRA, PIK3CA, PTEN, TP53 showed in some patients the coexistence of more than one variant (Supplementary Table 3). Four genes (ERBB2, FOXL2, MAP2K1, SRC) presented no mutations in any of the 89 cases analyzed and were excluded from further evaluation.

### Unsupervised hierarchical clustering based on patients’ genetic profile distinguished tumors with different grading

A hierarchical clustering analysis based on Euclidean distance between samples and Ward agglomerative procedure was applied to perform an unsupervised subdivision of the EC cohort taking into account only genetic characteristics. Variables considered were expressed as the number of mutations occurred in each gene (range 0 ÷ 4).

**Figure 1:**
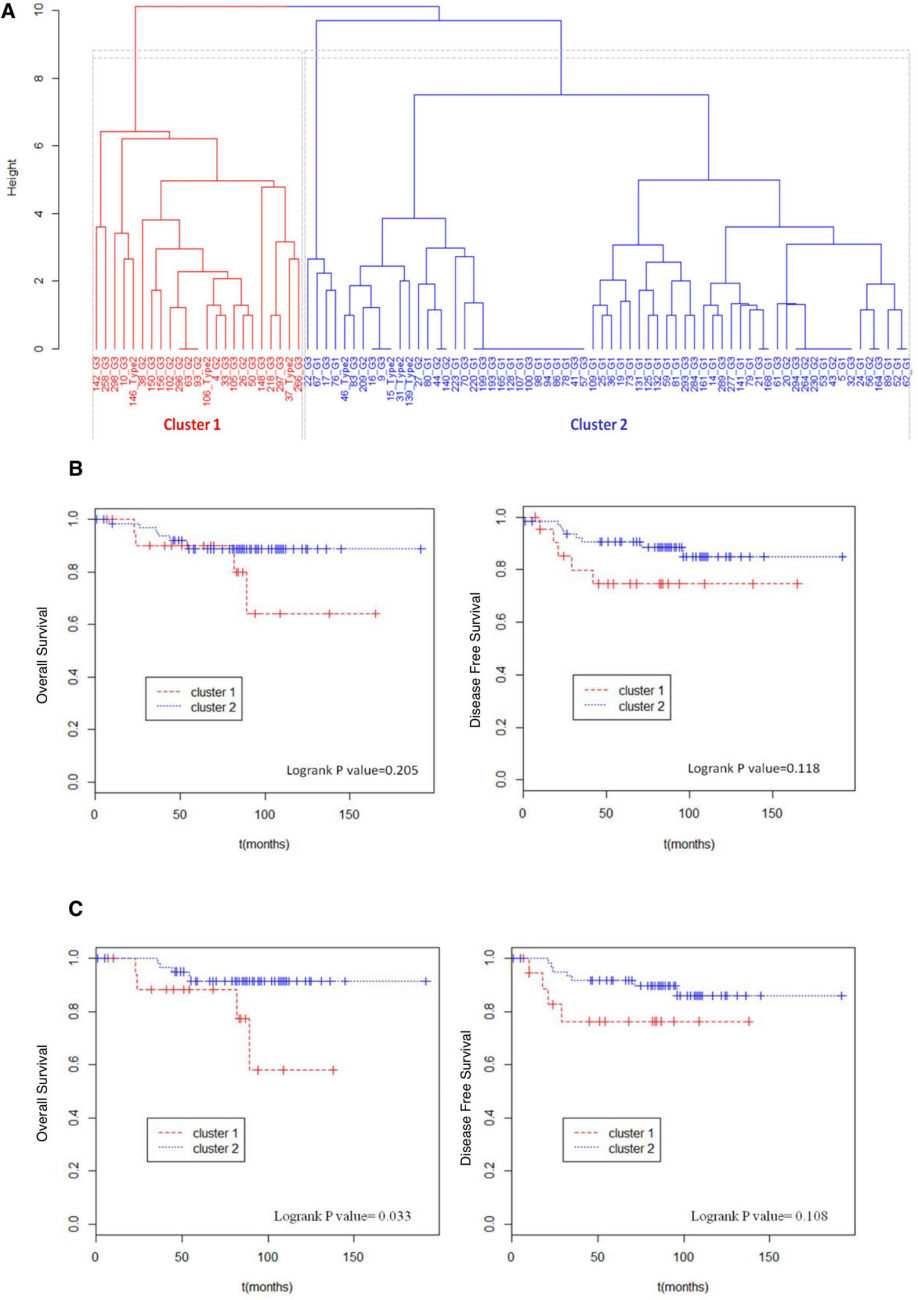
Unsupervised hierarchical clustering and survival analysis (**A**) Binary unsupervised hierarchical clustering performed on the total EC cohort. In x axis sample ID and relative tumor grade were reported, in y axis height expresses the distance between clusters. Cluster 1 and cluster 2 were respectively colored in red and blue. (**B**) Total EC cohort (89 patients) considered. Kaplan Meier curves were used to compare overall survival and disease free survival of patients in cluster 1 with those of patients in cluster 2. (**C**) Type 1 EC cohort (82 patients) considered. Kaplan Meier curves were used to compare overall survival and disease free survival of patients in cluster 1 with those of patients in cluster 2.

Two clusters derived from this analysis: Cluster 1 with 23 EC samples and Cluster 2 with 66 (Figure 1A). Table 1 summarizes the clinical features frequencies within the two clusters. Intriguingly, a strong association between clusters and tumor grading (*P* value < 0.001) was observed. In particular the molecular model efficiently identifies type 1 G1 tumors, positioning them all in cluster 2. Lax Kuman histological classification likewise resulted significantly associated with cluster subdivision; about 85% of the low grade tumors were grouped in cluster 2. By contrast, no differences in age, BMI, FIGO stage, lymph nodes positivity between the two clusters were observed.

**Table 1:** Distribution of clinical features within the two clusters

		CLUSTER		
		1 *N*, (%)	2 *N*, (%)	*P* value
**Tot**		23	66	
**Age**	64.8 ± 10.1	66.6 ± 11.7	64.1 ± 9.6	0.328
**BMI**	30.7 ± 8.4	31.8 ± 10.0	30.4 ± 7.9	0.523
**Grade**				***<0.001***
*G1*	33	0 (0.0)	33 (100.0)	
*G2*	16	7 (43.8)	9 (56.2)	
*G3*	33	13 (39.4)	20 (60.6)	
*Histotype 2*	7	3 (42.9)	4 (57.1)	
**Lax Kurman**				***0.037***
*Low*	49	7 (14.3)	42 (85.7)	
*High*	36	13 (36.1)	23 (63.9)	
*NA*	4	3	1	
**FIGO Stage**				0.522
*I -II*	74	18 (24.3)	56 (75.7)	
*III-IV*	15	5 (33.3)	10 (66.7)	
**Lymph node positivity**				1
*0*	80	21 (26.2)	59 (73.8)	
*At least 1*	9	2 (22.2)	7 (77.8)	
**Adjuvant therapy**				0.253
*No*	23	4 (17.4)	19 (82.6)	
*Yes*	44	15 (34.1)	29 (65.9)	
*NA*		4	18	

*P* value were calculated performing Fisher test.

### Molecular based clustering distinguished two groups of patients with different trend of survival

Next, we sought to investigate whether molecular clustering could be effective to distinguish patients with different prognosis. To this purpose, a Cox proportional hazard model was applied to compare the overall survival and the disease free survival between the two clusters.

At first, we performed the analysis over all 89 EC patients. Table 2 summarizes the number of events of death and recurrence registered in total population and reported the hazard ratio between the two clusters. The obtained differences between the two groups were not statistically significant (Figure 1B). However, due to the tumor type, where deaths and recurrence are quite rare, the changes in terms of recurrence between the two clusters (from 22% of cluster 1 to 14% of cluster 2) are indeed clinically relevant.

**Table 2:** Cox proportional hazard model for overall survival and disease free survival comparison between the 2 clusters

Total Population (89)					
		Patients	Events *N*, (%)	HR	Logrank *P* value
**Overall Survival**	Cluster 1	23	4 (17%)	-	-
	Cluster 2	66	7 (11%)	0.46	0.205
**Disease Free Survival**	Cluster 1	23	5 (22%)	-	-
	Cluster 2	66	9 (14%)	0.42	0.119

Histotype 1 Population (82)					
		Patients	Events *N*, (%)	HR	Logrank *P* value
**Overall Survival**	Cluster 1	20	4 (20%)	-	-
	Cluster 2	62	5 (8%)	0.26	***0.033***
**Disease Free Survival**	Cluster 1	20	4 (20%)	-	-
	Cluster 2	62	7 (11%)	0.38	0.108

Total population (89 patients) and only histotype 1 population were considered.

The same analysis was then performed on selected cases, composed of 82 type 1 ECs included in the genetic profiling (Figure 1C). When restricted to an histologically homogenous cohort, the Cox proportion hazard model demonstrated that the molecular clustering, inferred on the basis of the genetic profile, correlates significantly with patient’s disease specific survival (Logrank *P* value = 0.033). In particular, cluster 2 presented a 4 times lower risk of death because of the tumor (HR = 0.26) (Table 2). By contrast disease free survival probability was not significantly different between the two clusters (Logrank *P* value = 0.108).

Overall, these data seem to indicate that the molecular based clustering, proposed in this model, is suitable to distinguish “poor prognosis” EC patients (cluster 1) from “good prognosis” EC patients (cluster 2).

### Mutational status of a small group of genes influences tumor grading and patients prognostic classification

**Figure 2:**
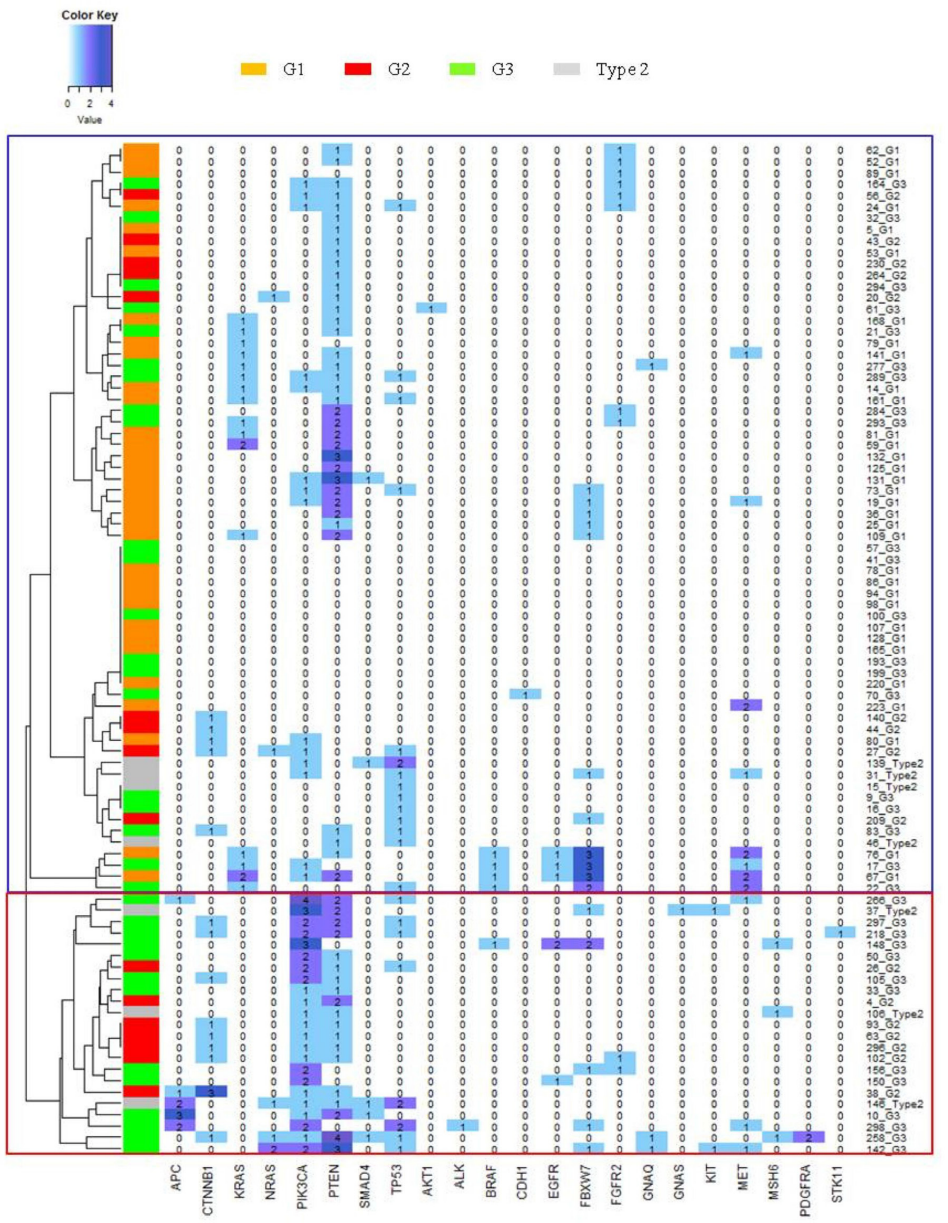
Gene mutations heatmap Y axis show clusters dendrogram, each row represent a patient and a color codify for the histological grade of the tumor. X axis reports the gene list. In each column the number of mutations of a gene in each samples are represented. Blue box identifies samples in cluster 2, while red box identifies samples in cluster 1.

In order to investigate which genes were the most relevant in this model and for the clustering of EC patients, we generated an heatmap to represent the number of mutations occurred in each gene for any single case (Figure 2).

Interestingly, heatmap representation shows that no mutations in APC gene were found in cluster 2 patients, while 9 mutations (corresponding to 5 out of 23 patients: 21.7%) were observed in cluster 1. By contrast, no mutations in KRAS were observed in cluster 1, while 14 patients in cluster 2 presented at least one KRAS mutation (14/66, 21.2%). Mutations in PIK3CA were observed in 23/23 (100%) patients in cluster 1 and 14/66 (21.2) in cluster 2, but all tumors presenting more than one variant for PIK3CA were localized in cluster 1. In cluster 1 19/23 (82.3%) patients presented both PIK3CA and PTEN mutation. In cluster 2 the coexistence of these mutated genes was observed only in 9/66 (13.6%) cases.

In order to statistically investigate these observations we analyzed the frequencies of mutations of each gene in the two clusters (Table 3). Statistical univariate analysis confirmed the significantly different distribution of APC, CTNNB1, KRAS, PIK3CA, PTEN, as observed in the heatmap. Furthermore, multivariate analysis confirmed a significant different distribution of CTNNB1 and PIK3CA mutations, suggesting a possible role of these gene mutational profiles as drivers of the cluster generation. In addition, total mutational load (calculated considering all 26 Trusight tumor genes) was found to be statistically different in the two clusters: while in “bad prognosis” cluster 1 a mean of 6 mutations for patient was observed, in cluster 2 the mean mutational load was only 2, suggesting as expected that the coexistence of a larger number of mutations could influence the development of a worse tumor phenotype.

**Table 3:** Univariate and multivariate statistical analysis of distribution of genes mutations in population clusters and tumor grades

		Clusters				Tumor Grading					
Gene mutations		1	2	*P*	Adjusted *P*	G1	G2	G3	Type 2	*P*	Adjusted *P*
**Total patients**	89	23	66			33	16	33	7		
**APC**				**<0.001**	**0.003**					**0.048**	0.744
**0**	84 (94.4)	18 (78.3)	66 (100.0)			33 (100.0)	15 (93.8)	30 (90.9)	6 (85.7)		
**1**	2 (2.2)	2 (8.7)	0 (0.0)			0 (0.0)	1 (6.2)	1 (3.0)	0 (0.0)		
**More than 1**	3 (3.4)	3 (13.0)	0 (0.0)			0 (0.0)	0 (0.0)	2 (6.1)	1 (14.3)		
**BRAF**				1	0.382					0.924	0.130
**0**	84 (94.4)	22 (95.7)	62 (93.9)			31 (93.9)	16 (100.0)	30 (90.9)	7 (100.0)		
**1**	5 (5.6)	1 (4.3)	4 (6.1)			2 (6.1)	0 (0.0)	3 (9.1)	0 (0.0)		
**More than 1**	0 (0.0)	0 (0.0)	0 (0.0)			0 (0.0)	0 (0.0)	0 (0.0)	0 (0.0)		
***CTNNB1***				***0.001***	***0.006***					0.742	0.420
***0***	65 (84.3)	14 (60.9)	61 (92.4)			32 (97.0)	8 (50.0)	28 (84.8)	7 (100.0)		
***1***	13 (14.6)	8 (34.8)	5 (7.6)			1 (3.0)	7 (43.8)	5 (15.2)	0 (0.0)		
***More than 1***	1 (1.1)	1 (4.3)	0 (0.0)			0 (0.0)	1 (6.2)	0 (0.0)	0 (0.0)		
***EGFR***				0.298	0.786					0.710	0.398
***0***	84 (94.4)	21 (91.4)	63 (95.5)			31 (93.9)	16 (100.0)	30 (90.9)	7 (100.0)		
***1***	4 (4.5)	1 (4.3)	3 (4.5)			2 (6.1)	0 (0.0)	2 (6.1)	0 (0.0)		
***More than 1***	1 (1.1)	1 (4.3)	0 (0.0)			0 (0.0)	0 (0.0)	1 (3.0)	0 (0.0)		
***FBXW7***				0.787	0.317					0.905	0.426
***0***	73 (82.0)	18 (78.3)	55 (83.3)			26 (78.8)	15 (93.8)	27 (81.8)	5 (71.4)		
***1***	11 (12.4)	4 (17.4)	7 (10.6)			5 (15.2)	1 (6.2)	3(9.1)	2 (28.6)		
***More than 1***	5 (5.6)	1 (4.3)	4 (6.1)			2 (6.0)	0 (0.0)	3 (9.1)	0 (0.0)		
***FGFR2***				1	0.409					0.608	0.714
***0***	79 (88.8)	21 (91.3)	58 (87.9)			29 (87.9)	14 (87.5)	29 (87.9)	7 (100.0)		
***1***	10 (11.2)	2 (8.7)	8 (12.1)			4 (12.1)	2 (12.5)	4 (12.1)	0 (0.0)		
***More than 1***	0 (0.0)	0 (0.0)	0 (0.0)			0 (0.0)	0 (0.0)	0 (0.0)	0 (0.0)		
***KRAS***				***0.021***	0.222					***0.043***	0.348
***0***	73 (82.0)	23 (100.0)	50 (75.8)			23 (69.7)	16 (100.0)	27 (81.8)	7 (100.0)		
***1***	14 (15.7)	0 (0.0)	14 (21.2)			8 (24.2)	0 (0.0)	6 (18.2)	0 (0.0)		
***More than 1***	2 (2.3)	0 (0.0)	2 (3.0)			2 (6.1)	0 (0.0)	0 (0.0)	0 (0.0)		
***MET***				0.322	0.380					0.612	0.183
***0***	78 (87.6)	20 (87.0)	58 (87.8)			28 (84.8)	16 (100.0)	28 (84.8)	6 (85.7)		
***1***	7 (7.9)	3 (13.0)	4 (6.1)			2 (6.1)	0 (0.0)	4 (12.1)	1 (14.3)		
***More than 1***	4 (4.5)	0 (0.0)	4 (6.1)			3 (9.1)	0 (0.0)	1 (3.1)	0 (0.0)		
***NRAS***				0.106	0.192					0.151	0.583
***0***	84 (94.4)	20 (87.0)	64 (97.0)			33 (100.0)	14 (87.5)	31 (94.0)	6 (85.7)		
***1***	4 (4.5)	2 (8.7)	2 (3.0)			0 (0.0)	2 (12.5)	1 (3.0)	1 (14.3)		
***More than 1***	1 (1.1)	1 (4.3)	0 (0.0)			0 (0.0)	0 (0.0)	1 (3.0)	0 (0.0)		
***PIK3CA***				***<0.001***	***<0.001***					***<0.001***	**0.009**
***0***	52 (58.4)	0 (0.0)	52 (78.8)			26 (78.8)	7 (43.8)	17 (51.5)	2 (28.6)		
***1***	25 (28.1)	11 (47.8)	14 (21.2)			7 (21.2)	8 (50.0)	6 (18.2)	4 (57.1)		
***More than 1***	12 (13.5)	12 (52.2)	0 (0.0)			0 (0.0)	1 (6.2)	10 (30.3)	1 (14.3)		
***PTEN***				***0.049***	0.264					0.503	0.251
***0***	33 (37.1)	4 (17.4)	29 (43.9)			12 (36.4)	4 (25.0)	14 (42.4)	3 (42.9)		
***1***	36 (40.4)	11 (47.8)	25 (37.9)			11 (33.3)	11 (68.8)	11 (33.3)	3 (42.9)		
***More than 1***	20 (22.5)	8 (34.8)	12 (18.2)			10 (30.3)	1 (6.2)	8 (24.3)	1 (14.2)		
***SMAD4***				0.106	0.594					0.057	0.743
***0***	84 (94.4)	20 (87.0)	64 (97.0)			32 (97.0)	16 (100.0)	31 (93.9)	5 (71.4)		
***1***	5 (5.6)	3 (13.0)	2 (3.0)			1 (3.0)	0 (0.0)	2 (6.1)	2 (28.6)		
***More than 1***	0 (0.0)	0 (0.0)	0 (0.0)			0 (0.0)	0 (0.0)	0 (0.0)	0 (0.0)		
***TP53***				0.186	0.153					***<0.001***	**0.024**
***0***	67 (75.3)	15 (65.2)	52 (78.8)			30 (90.9)	13 (81.2)	22 (66.7)	2 (28.6)		
***1***	19 (21.3)	6 (28.1)	13 (19.7)			3 (9.1)	3 (18.8)	10 (30.3)	3 (42.8)		
***More than 1***	3 (3.4)	2 (8.7)	1 (1.5)			0 (0.0)	0 (0.0)	1 (3.0)	2 (28.6)		

Finally, we explored the correlation between genes mutations and EC tumor grade (Table 3). In a univariate analysis KRAS, PIK3CA and TP53 mutations presented a frequency distribution significantly different among the distinct tumor grades. In particular KRAS mutations were more recurrent in low grade type 1 EC (*p* = 0.043) while single or double mutations of PIK3CA and TP53 occurred with higher frequency in high grade EC (*p* = <0.001). The specific association of PIK3CA and TP53 mutations and tumor grade was also confirmed in the multivariate analysis.

### A data mining tool based on few genes mutation analysis could support the prognosis of EC patients

All together, these data indicate that mutation analysis in a limited number of genes could generate a model to improve risk-based stratification of EC patients. In order to provide the clinician with an easy and useful tool for the EC patients prognostic classification, we used a data mining approach to define a consecutive sequence of rules and to generate a schematic representation of the model.

**Figure 3:**
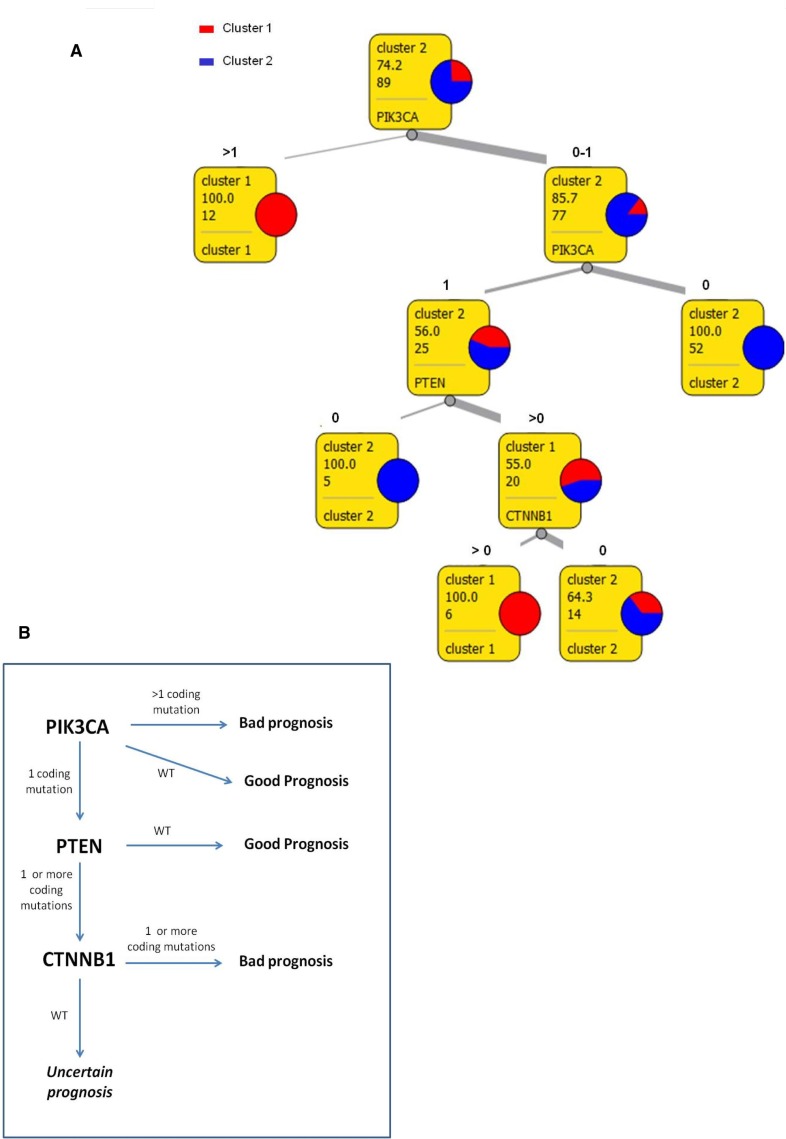
Schematic decision model (**A**) Decision tree for tumors classification in two prognostic clusters. In squares: first row reports the majority class, second row expresses the frequency of the majority class, third row reports the number of instances considered in that leaf, fourth row shows the class of destination or the next attribute that should be evaluated. (**B**) Flowchart of the molecular model for EC risk stratification.

Figure 3A shows a classification tree that was created to summarize principal rules that drove patients clusterization. PIK3CA, PTEN and CTNNB1 mutational status appears to be the main drivers in cluster generation. In particular, patients presenting more than one mutation in PIK3CA are predicted to have a bad prognosis (cluster 1) while patients with no mutation in PIK3CA can be automatically classified in good prognosis group (cluster 2). Instead, in patients with only one mutation in PIK3CA, the evaluation of PTEN and CTNNB1 will be necessary: coexistence of mutations in PIK3CA, PTEN and CTNNB1 can be considered a marker of bad prognosis (cluster 1) while women with one mutation on PIK3CA but no mutation in PTEN are predicted to have a better survival (cluster 2). A 10-fold cross validation was used to evaluate this data mining method. The decision tree proposed above had 90% classification accuracy, 76% Matthew Correlation Coefficient, 74% sensitivity and 97% specificity.

However, 14 out of 89 patients were not classified in accordance with the proposed model, suggesting the need of improving the sensibility of this tool through the analysis of additional mutational hotspots.

## DISCUSSION

To date histological characterization and staging are the gold standard for EC prognosis. Different tumor histological criterion such as Bockman typing, FIGO stage, grading and Lax Kurman binary classification [3–5, 11, 12] can be used to predict EC outcome. Nevertheless, in some morphologically intermediate and doubtful cases, anatomo-pathological classification and risk based stratification turns out to be insufficient and inefficient.

In this study we explored the mutational profile of a selected cohort of EC with the aim of developing a simple genetic-based tool to improve the accuracy of the current stratification methods for EC patients. We investigated the occurrence of mutations in a panel of 26 cancer related genes, in a population of 89 EC with different histological characteristics and different outcome. An unsupervised hierarchical clustering analysis demonstrated that the mutational profiles obtained from this analysis effectively separate endometrial tumors in two groups characterized by different tumor grades and different prognosis.

Statistical analysis were performed to define which of the genes investigated could be considered principal drivers of the prognostic clustering: APC, CTNNB1, PIK3CA, PTEN, SMAD4 and TP53 resulted as the most influencing mutated genes. Moreover, rules definition indicates that not only the presence or absence of somatic and damaging mutations on these genes, but also the number of variants occurred on the same gene in each sample can be determinant in predicting patient outcome. Finally a data mining strategy based on the generation of a decision tree was used to summarize a consequential list of classification rules applicable to perform EC risk stratification based only on molecular data (Figure 3B).

The PI3K pathway activation regulates key aspects of cancer biology including metabolism, cellular growth, survival and resistance to apoptosis [13]. PTEN counteracts the activation of PI3K pathway by hydrolyzing and inactivating phosphatidylinositol 3,4,5- triphosphate (PIP3), the molecule responsible for the activation of the signalling cascade [14]. The PI3K/AKT/mTOR pathway is also involved in cross-talk with other signalling pathways, including the RAS/RAF/MEK [15] and estrogen receptor (ER) [16, 17]. Data from the literature, indicate that constitutive activation of the PI3K/AKT pathway in EC occurs mostly through mutational inactivation of PTEN or by mutational activation of PIK3CA [18]. A high frequency of PIK3CA and PTEN mutation and often coexistence of mutations in both these genes have already been described as frequently occurring in EC [19]. Interestingly, in our analysis, PIK3CA and PTEN mutations were identified as the principal determinants of patients prognostic clustering further highlighting the fundamental role of this pathway in EC. Moreover we showed that two PIK3CA mutations or the coexistence of PIK3CA and PTEN mutations are needed to influence endometrial cancer prognosis. Our observations, in accordance with data presented by Oda *et al.* [19] that described the lack of influence of a single PIK3CA mutation on EC, indicated that in EC more than one mutational event in PI3K/AKT pathway genes is necessary to functionally influence this pathway and to induce a constitutively activated cascade fostering tumor aggressiveness.

Given the frequency of abnormalities in the PI3K/AKT pathway, this signaling pathway represents one of the most promising targets for EC therapy. Thus, the identification of genetic mutations within key genes of this pathway could represent valuable markers for patient selection and therapy response monitoring.

The third gene involved in the proposed prediction model was CTNNB1. For its high mutation frequency, the role of CTNNB1 mutations has been often investigated in association with EC. In particular, mutations occurred in CTNNB1 exon 3 as in our cases were associated with an accumulation of B-catenin in nucleus [20] and with a consequent activation of Wnt/β-catenin pathway that was already associated with worse survival in type 1 EC [21]^.^ The same observation on CTNNB1 were reported in a recent work that showed an association between CTNNB1 mutation in low grade EC patients and a higher risk of tumor recurrence [22].

The method described in this work will need to be corroborated in separates sets of ECs and with a bigger cohort of patients to strengthen the prognostic differences obtained with our model. These data suggest that the approach described by this work could became a double function tool. First of all it represents an easy and relatively economic molecular profiling of EC that could be associated to histological classification to make patients prognosis in particular in doubtful and intermediate cases. Secondly, the development of a small NGS panel based on the mutational analysis of the few genes emerged from this model could represent a rapid method to investigate those genes that are considered the most promising molecular target for EC therapy.

Currently, in the most recent guidelines, some management recommendations for EC patients such as use of adjuvant therapy are still based on scant evidences [23]. The genetic-based model proposed in our study could provide a more appropriated and tailored treatment to patients diagnosed with EC. In particular, this clustering strategy could help to identify a genetic subgroup of patients that would benefit for adjuvant therapy and closer follow-up but that based on the current classification remain undertreated. Moreover, the application of this tool could help sparing low risk EC patients from aggressive therapy and intensive follow-up.

## MATERIALS AND METHODS

### Patients selection

Clinical annotations of EC patients treated and followed at the Azienda USL -IRCCS di Reggio Emilia (Italy) from 2000 to 2016 were checked for cohort selection. Patients with histological diagnosis of type I and type II EC who received surgery were included in the study protocol. Exclusion criteria were: inadequate EC management according to internal and international guidelines [24, 25], neoadjuvant chemotherapy performed before surgery, less than 18 years of age, non-Caucasian ancestry, inadequate follow-up according to internal guidelines, absence of written informed consent, diagnosis of a previous or concurrent cancer(s) and unavailable follow-up data. A follow-up was defined “adequate” in case of adherence to the following schedule: type I EC at stage IA and grading G1/G2 - physical and gynecological examination, and transvaginal ultrasound every 6 months for the first 2 years, then every 12 months for at least 3 years; type I EC at stage IB and/or any grading G3 and any type II tumor - physical and gynecological examination, and transvaginal ultrasound every 6 months for the first 5 years. Further investigations such as abdominal ultrasound, chest X-ray, computed tomography scan, and serum CA 125 levels were performed if clinically indicated. 105 patients responded to inclusion criteria and were considered for the study. Eighty-nine out of 105 patients had FFPE tumor tissue useful for genetic analysis.

Clinical, pathological and genetic data of every patients remained anonymous and were recorded in an electronic password-protected database. The study was approved by the Local Ethical Committee and all patients provided written informed consent to take part to the study.

### Next generation sequencing

DNA was extracted from Formalin fixed paraffin embedded (FFPE) EC tissues using Maxwell nucleic acid extractor (Promega) and then quantified and quality evaluated using Kapa SYBR Fast qPCR kit.

Trusight tumor 26 kit (Illumina) was used for libraries preparation and sequencing was performed on Miseq V2 cartridge 300 cycles (2x121).

MiSeq Reporter software was used to elaborate MiSeq row data and produce fastq and vcf files. Variant studio software (Illumina) was used to visualize list of mutations occurred in each sample, annotate them and apply selection filters. Mutation were considered reliable if presenting a minimum frequency of 5% and a minimum coverage of 500×.

### Statistical analysis

All analysis performed in this study were elaborated using R software.

To generate the unsupervised hierarchical clustering it was taken into account the number of non-silent mutations occurred in each gene for each patient. Variables were expressed as ordinal values with a range from 0 (no mutation) to 4. Only data obtained from sequencing were used as attributes in the analysis, no clinical variables were included. Euclidean distance was used to compute distance measures between samples. Ward agglomerative hierarchical clustering procedure was applied. A two clusters subdivision was chosen to obtain numerically comparable groups.

Analysis of association between clusters, clinical characteristics and gene mutations were performed using Fisher test and generalized linear models. Survival analysis was conducted applying Cox proportional hazard model and Kaplan Meier curves were generated. Associations and differences were considered statistically significant if presented a *P* value lower than 0.05

### Classification tree generation

Orange Canvas software [26] was used to generate the classification tree. Mutational status of genes were the only attributes considered in the analysis and “cluster 1” and “cluster 2” were the two decision class. All attributes were defined as continuous. Gain ratio was used as attribute selection criterion. For pre-pruning, a minimum of 5 instances for leave was fixed. For post-pruning the recursively merging of leaves with the same majority class was performed and m parameter was fixed to 1. Classification accuracy, sensitivity and specificity of these method were calculated after a 10-fold cross-validation.

## SUPPLEMENTARY MATERIALS TABLES



## Abbreviations

EC: Endometrial Cancer
OS: Overall Survival
FIGO: International Federation of Gynaecology and Obstetrics
LVSI: lymphovascular space invasion
NGS: Next Generation Sequencing
TCGA: The Cancer Genome Atlas
FFPE: Formalin fixed paraffin embedded
