# Supplementary material for: Computational development of a molecular-based approach to improve risk stratification of endometrial cancer patients
Source: Oncotarget. 2018 May 22;9(39):25517–28. doi: 10.18632/oncotarget.25354 (PMC5986657; doi:10.18632/oncotarget.25354)
Supplement: Supplementary file 1 [file oncotarget-09-25517-s001.pdf]

Computational development of a molecular-based approach to improve risk stratification of endometrial cancer patients

SUPPLEMENTARY MATERIALS

Supplementary Table 1: Clinical and pathological characteristics of the 89 patients included in the study

	Total patients 89 (*n*, %)
**Age (mean, range)**	64.6 (42 ÷ 85)
**BMI (mean, range)**	31.8 ± 10.5
**Diabetes**	
No	67 (75.3)
Yes	19 (21.3)
NA	3 (3.4)
**Ipertension**	
No	36 (40.4)
Yes	50 (56.2)
NA	3 (3.4)
**Histotype**	
Type 1	82 (92.1)
Type 2	7 (7.9)
**Type 1_Grade (82)**	
G1	33 (40.2)
G2	16 (19.6)
G3	33 (40.2)
**FIGO_stage**	
IA	13 (14.6)
IB	36 (40.5)
IC	17 (19.1)
II	8 (9.0)
IIIA	5 (5.6)
IIIB	1 (1.1)
IIIC	8 (9.0)
IV	1 (1.1)
**Death because of the tumor**	11 (12.4)
**Recurrence**	14 (15.7)
**Follow up (mean, range)**	79 (1–192)

Supplementary Table 2: Numeric report of all 893 genetic variants identified by NGS

	893 Genetic variants identified											
	285 Genetic variants included in the analysis						608 Genetic variants excluded from the analysis					
	Missense variants	Frameshift variants	Splice acceptor variants	Splice donor variants	Stop gain variants	Tot	Synonymous variants	3 prime UTR variants	Polymorphic Missense variants	Intron variants	Non coding exon variants	Tot
AKT1	1	0	0	0	0	1	0	0	0	0	0	0
ALK	1	0	0	0	0	1	0	0	0	0	0	0
APC	2	2	0	0	5	9	78	0	0	0	0	78
BRAF	5	0	0	0	0	5	0	0	0	0	0	0
CDH1	1	0	0	0	0	1	8	0	0	0	0	8
CTNNB1	16	0	0	0	0	16	0	0	0	0	0	0
EGFR	6	0	0	0	0	6	2	0	0	1	66	69
ERBB2	0	0	0	0	0	0	2	0	0	0	0	2
FBXW7	19	0	0	0	5	24	1	0	0	0	0	1
FGFR2	10	0	0	0	0	10	1	0	0	0	0	1
FOXL2	0	0	0	0	0	0	0	0	0	0	0	0
GNAQ	3	0	0	0	0	3	0	0	0	46	0	46
GNAS	1	0	0	0	0	1	0	0	0	0	0	0
KIT	2	0	0	0	0	2	27	0	0	0	0	27
KRAS	18	0	0	0	0	18	0	35	0	0	0	35
MAP2K1	0	0	0	0	0	0	0	0	0	0	0	0
MET	14	0	1	0	0	15	128	0	3	0	0	131
MSH6	3	0	0	0	0	3	1	0	0	0	0	1
NRAS	6	0	0	0	0	6	0	0	0	0	0	0
PDGFRA	2	0	0	0	0	2	113	0	0	0	0	113
PIK3CA	53	0	0	0	0	53	4	0	0	0	0	4
PTEN	40	17	3	2	16	78	4	0	0	2	0	6
SMAD4	5	0	0	0	0	5	0	0	0	0	0	0
SRC	0	0	0	0	0	0	1	0	0	0	0	1
STK11	1	0	0	0	0	1	0	0	0	0	0	0
TP53	19	2	0	0	4	25	9	0	76	0	0	85

Only 285 somatic variants supposed to have effect on proteins functions were included in further analysis.

Supplementary Table 3: Summary of somatic mutations alleged to have effect on protein function and included in further analysis

Gene	Total number of mutation	Number of mutated patients	HGVSc
AKT1	1	1	c.142C>T
ALK	1	1	c.3521T>C
APC	9	5	c.3925G>T, c.4630G>T, c.4729G>T, c.2626C>T, c.4661delA, c.4738A>G, c.2677G>A, c.4385_4386delAG
BRAF	5	5	c.1328G>T, c.1805C>A
CDH1	1	1	c.1073C>T
CTNNB1	16	14	c.94G>C, c.122C>T, c.94G>A, c.101G>A, c.121A>G, c.101G>T, c.134C>T, c.98C>G, c.110C>T, c.100G>A, c.97T>G
EGFR	6	5	c.2505C>A, c.2505C>A, c.2491C>T, c.2258C>T, c.2591C>T
FBXW7	24	16	c.1660G>T, c.1719C>A, c.2009G>T, c.2065C>T, c.1660G>T, c.1513C>T, c.2066G>A, c.1345G>T, c.1436G>A, c.1634A>T, c.1393C>T, c.1694T>G, c.1552G>A, c.1268G>T, c.1394G>A
FGFR2	10	10	c.755C>G
GNAQ	3	3	c.524C>T, c.803C>T, c.562G>T
GNAS	1	1	c.2524C>T
KIT	2	2	c.1652C>A, c.1444G>A
KRAS	18	16	c.35G>T, c.35G>C, c.35G>A, c.312G>T, c.38G>A, c.35G>T, c.34G>T
MET	15	11	c.1586G>T, c.638C>T, c.1586G>T, c.3817C>A, c.4036C>A, c.3314-1G>T, c.901A>G, c.3029C>T, c.1688C>T, c.2962C>T, c.504G>T
MSH6	3	3	c.3232G>T, c.3319G>T, c.3388G>A
NRAS	6	5	c.191A>G, c.235C>A, c.405G>T, c.181C>A, c.35G>A, c.122G>A
PDGFRA	2	1	c.1780G>T, c.1921C>T
PIK3CA	53	37	c.263G>A, c.1337G>T, c.1634A>G, c.302_304delTAA, c.3140A>G, c.3143A>G, c.113G>A, c.112C>T, c.3139_3140delCAinsAT, c.3010A>G, c.333G>C, c.3104C>T, c.1258T>C, c.3169T>C, c.241G>A, c.1345C>T, c.419G>A, c.277C>T, c.329_331delAAA, c.1351G>A, c.3062A>G, c.3073A>G, c.278G>A, c.23G>A, c.1634A>C, c.1633G>A, c.1625A>C, c.317G>T, c.353G>A, c.1624G>A
PTEN	81	56	c.748delT, c.389G>A, c.462C>A, c.388C>T, c.361G>A, c.193T>G, c.395G>A, c.528delT, c.388C>G, c.64_69delGACTTA, c.517C>T, c.697C>T, c.224_228delATTAT, c.217_218insA, c.406T>C, c.403A>G, c.1031_1040delAGCTGTACTT, c.289C>T, c.511C>T, c.601G>T, c.794_795insA, c.380G>A, c.518G>A, c.94_96delATT, c.635-1G>A, c.740_741insA, c.253+1G>T, c.697_700delCGAC, c.274G>T, c.295G>T, c.19G>T, c.16A>G, c.100G>A, c.493G>A, c.389G>T, c.217G>T, c.634+1G>T, c.85_101delTATCCAAACATTATTGC, c.795delA, c.179A>C, c.431A>C, c.677C>T, c.237_238insA, c.464A>G, c.165-2A>G, c.631_632insG, c.665_678delTGAAGATATATTCC, c.37A>T, c.746T>G, c.635-1G>C, c.511C>A, c.610delC, c.389delG, c.385G>A, c.476G>T, c.323T>A
SMAD4	5	5	c.1544G>T, c.1612G>A, c.1609G>T, c.1487G>A
STK11	1	1	c.929G>A
TP53	25	22	c.659A>G, c.610G>T, c.1091C>A, c.645T>A, c.817C>T, c.659A>G, c.523C>T, c.359A>C, c.475G>A, c.637C>T, c.365_366delTG, c.524G>A, c.800G>A, c.380C>T, c.140delC, c.799C>T, c.742C>T, c.743G>A, c.452C>G, c.993G>T
